# Simple and rapid sample preparation system for the molecular detection of antibiotic resistant pathogens in human urine

**DOI:** 10.1007/s10544-016-0031-9

**Published:** 2016-02-04

**Authors:** Martha Valiadi, Sumit Kalsi, Isaac G. F. Jones, Carrie Turner, J. Mark Sutton, Hywel Morgan

**Affiliations:** Electronics and Computer Science, University of Southampton, Southampton, SO17 1BJ UK; Institute for Life Sciences, University of Southampton, Southampton, SO17 1BJ UK; National Infections Service, Public Health England, Porton Down, Salisbury, SP4 0JG UK

**Keywords:** Antibiotic resistant bacteria, Magnetic beads, Microfluidics, Recombinase polymerase amplification, Sample preparation, Urine

## Abstract

Antibiotic resistance in urinary tract infections (UTIs) can cause significant complications without quick detection and appropriate treatment. We describe a new approach to capture, concentrate and prepare amplification-ready DNA from antibiotic resistant bacteria in human urine samples. *Klebsiella pneumoniae* NCTC13443 (*bla*_CTX-M-15_ positive) spiked into filtered human urine was used as a model system. Bacteria were captured using anion exchange diaethylaminoethyl (DEAE) magnetic microparticles and concentrated 200-fold within ~3.5 min using a custom, valve-less microfluidic chip. Eight samples were processed in parallel, and DNA was released using heat lysis from an integrated resistive heater. The crude cell lysate was used for real time Recombinase Polymerase Amplification (RPA) of the *bla*_CTX-M-15_ gene. The end to end processing time was approximately 15 min with a limit of detection of 1000 bacteria in 1 mL urine.

**Electronic supplementary material:**

The online version of this article (doi:10.1007/s10544-016-0031-9) contains supplementary material, which is available to authorized users.

## Introduction

Antibiotic resistance is a worldwide public health concern. As pathogens evolve new antibiotic resistance mechanisms, even simple infections will become difficult to treat (O’Neill 2014). Rapid detection of antibiotic resistant pathogens is key for targeted and effective patient treatment and to limit the development of further resistance caused by the use of ineffective or broad-spectrum antibiotics. Current routine clinical tests to detect antibiotic resistant bacteria rely on time-consuming cell culture, or on molecular detection by polymerase chain reaction (PCR) based approaches that require dedicated laboratories and expert users. Therefore, rapid and sensitive Point-of-Care (PoC) tests are critical to enable directed diagnosis of antibiotic resistant pathogens as the basis for prescribing practice.

Urinary tract infections (UTIs) are common bacterial infections that account for substantial health costs and morbidity (Abbo and Hooton 2014). *Klebsiella pneumoniae* and other extended spectrum β-lactamase (ESBL) and carbapenemase producing Enterobacteriaceae have emerged as a major healthcare issue in the last 10–20 years (Pitout et al. 2015). Resistance to extended spectrum β-lactam and carbapenem antibiotics have been classed as a serious hazard in the USA where 26,000 cases were reported in 2013, causing 1700 deaths (Control and Prevention 2013). The CTX-M enzyme family is the most prevalent amongst the wide range of ESBL enzymes, conferring resistance to key β-lactam antibiotics (Bonnet 2004; D’Andrea et al. 2013; Zhao and Hu 2013) in at least 26 bacterial species, residing in both nosocomial and community environments (Zhao and Hu 2013). Therefore, a gene encoding for the widespread CTX-M-15 isoform, *bla*_CTX-M-15_, which encodes a protein with enhanced catalytic activity (Zhao and Hu 2013), is an ideal candidate for demonstrating rapid PoC detection system for antibiotic resistant pathogens.

Significant advances in both highly sensitive and rapid (<20 min) isothermal DNA amplification techniques (reviewed by Niemz et al. 2011), as well as their implementation on easy automatable and miniaturized microfluidic platforms, have resulted in promising developments in PoC bacterial detection systems. For example, rolling circle amplification (RCA) has been integrated with microfluidic chips to detect *Salmonella* (Sato et al. 2010) and *Pseudomonas* (Kuhnemund et al. 2014). Loop-mediated isothermal amplification (LAMP) has been performed on various microfluidic platforms, including polymer chips (Tourlousse et al. 2012), PDMS chips (Fang et al. 2010; Wang et al. 2011) and centrifugal lab-on-a-disc platform (Kim et al. 2014) to detect a variety of human, water and food borne bacteria and viruses. Recombinase polymerase amplification (RPA) has also been implemented on micro-devices. This is an isothermal DNA amplification technique with distinct advantages including a lower incubation temperature, ease of primer design, high specificity and sensitivity for the target gene. RPA uses two oligonucleotide primers to prime the enzymatic amplification of a DNA fragment of interest, while its quantity can be monitored using fluorescent probes. The utility of this technology has now been demonstrated for the detection of a variety of pathogens (Euler et al. 2013; Ahmed et al. 2014; Kersting et al. 2014a, b; Xia et al. 2015), including real-time RPA on microfluidic devices (Lutz et al. 2009; Kalsi et al. 2015; Tortajada-Genaro et al. 2015; Tsaloglou et al. 2015). Most importantly, RPA has a high tolerance to crude samples, for example undiluted human urine (Krolov et al. 2014), without the need for extensive purification steps.

Despite significant progress in microfluidic based DNA detection assays, the development of a complete “sample in-answer out” device for PoC tests still faces some important obstacles. A key pre-requisite in any microfluidic PoC molecular diagnostic system is sample pre-processing to interface real clinical samples of several mL to the μL to nL volumes used in microfluidic molecular assays (Cui et al. 2015). An ideal sample preparation system should include a simple cell capture and pre-concentration unit as well as a DNA preparation method. Sample pre-concentration for pathogen analysis is arguably the most challenging part of a DNA-based assay. One of the simplest methods for capturing bacteria in solution is the use of small magnetic beads with various coatings. This approach can be easily incorporated into a passive sample preparation device, and requires no complex fluidics. Antibody coated beads attach to surface antigens of specific bacteria and can be used to separate them from a mixed community for detection by fluorescence microscopy (Wen et al. 2013), PCR (Beyor et al. 2009), loop mediated isothermal amplification (Wang et al. 2011) or transcriptome studies (Dai et al. 2011). Aptamers can also be used instead of antibodies as the capture molecule (Suh et al. 2014). However, some of these approaches have significant limitations such as requiring the bacteria to be in a particular buffer to facilitate capture, or requiring a nucleic acid extraction after cell capture (Dai et al. 2011; Suh et al. 2014), in addition to the time and financial burden of producing the capture molecules. Furthermore, antibody approaches are limited to only a single known target organism, which has already been isolated and cultured, and is assumed to represent the wild-type strains. Such detection approaches that target specific organisms instead of the genes responsible for antibiotic resistance, might miss species with emerging resistance (i.e. those that contain a newly acquired resistance gene). All these issues make antibody and aptamer coated capture beads unsuitable for bacterial community-level multiplex analysis.

An alternative, yet little-explored approach to capture and concentrate microbes from liquid samples is ion-exchange magnetic beads. Yang et al. (2011) showed how anion-exchange diethylaminoethyl (DEAE) coated magnetic beads could improve the detection limit of a PCR-based assay for *Escherichia coli* (Gram-negative) and *Agrobacterium tumefaciens* (Gram-positive) by 2–3 orders of magnitude compared to that without pre-concentration of the organisms. In different work, it was found that DEAE anion-exchange were superior to another four types of ion exchange beads, including beads coated with polyaspartic acid (PAA), giving the highest capture efficiency for *E. coli*, *Enterococcus* spp. and *Salmonella* spp. (Guo et al. 2009). However, the compatibility of this method with DNA amplification protocols was not investigated. Importantly, in both studies the protocols were developed to isolate bacteria suspended in water, which is certainly not representative of clinical samples. While surface functionalized magnetic beads show great promise for bacterial capture and pre-concentration, none of this work has been performed with clinical samples, nor has the potential for direct interfacing (i.e. without purification or washing) with DNA amplification been explored.

Bacteria in solution are usually concentrated using lab scale centrifugation, which has also been implemented on a microscale using microstructures fabricated on a compact disc (CD) (Cho et al. 2014; Kong et al. 2015). More complex cell concentration methods involve manipulating the flow of cells using size or charge based differentiation methods (Pratt et al. 2011). Simpler cell capture approaches using a physical substrate, such as a filter, (Baier et al. 2009; Gulliksen et al. 2012; Gan et al. 2014; Zhuang et al. 2015a, b) or beads (Cho et al. 2007; Ritzi-Lehnert et al. 2011) have also been demonstrated on microfluidic platforms. However, these methods require electrical power to control the sample flow or the magnetic beads. Other approaches for sample preparation involve cell lysis followed by DNA concentration using solid phase extraction on microdevices (Kulinski et al. 2009; Mahalanabis et al. 2009, 2010; Van Heirstraeten et al. 2014; Sun et al. 2015). In microfluidic systems, cell lysis is often achieved chemically using varying combinations of surfactants, enzymes and chaotropic agents (Jebrail et al. 2014), however, this necessitates a variety of chambers, reagents and control structures to be built into the device. Thermal methods, where cells are lysed at high temperature to release DNA, are popular due to their simplicity of implementation (Marshall et al. 2012).

In this study, we describe a simple streamlined method to rapidly capture, pre-concentrate and lyse bacterial pathogens from human urine for subsequent detection of antibiotic resistance genes. We simulate clinical samples by adding bacteria to filtered human urine, demonstrating a proof-of-concept sample processing protocol. This includes capture of bacteria from human urine using anion-exchange magnetic beads, concentration of the sample from 1 mL into small (~6 μL) volumes using a using an 8 channel, valve-less magnetic microfluidic device in just 3.5 min, and an optional heat lysis on the device to provide amplification-ready DNA. The simple sample processing method is coupled with a highly robust DNA amplification technique, Recombinase Polymerase Amplification (RPA). The protocol does not include any washing steps or buffer replacement for DNA purification. Combining this sample preparation approach with RPA of the *bla*_CTX-M-15_ gene, has led to a rapid assay for antibiotic resistance detection, with a sample preparation time of 10 min and RPA run time of 20 min for a highly sensitive of detection 1000 bacteria colony forming units. We demonstrate the utility of this method in human urine samples spiked with antibiotic resistant *Klebsiella pneumoniae*, thus proving its potential clinical relevance.

## Materials and methods

### Bacterial culture

The target organism used to develop this test was *Klebsiella pneumoniae* NCTC 13443, isolated from a human clinical sample and obtained from the National Collection of Type Cultures at Public Health England (PHE). The genome of this strain has been sequenced (EBI accession number SAMEA2742597) and is known to contain genes for the ESBL CTX-M-15 and the carbapenemase NDM-1. Bacteria were maintained on Tryptone Soy Agar (TSA) plates. To ensure healthy growth, bacteria were transferred onto new plates biweekly and following an overnight incubation at 37 °C, they were stored at 4 °C. To produce liquid cultures for experiments, a bacterial colony was transferred from a plate into 15 mL of Tryptone Soy Broth (TSB) medium and the culture was incubated at 37 °C overnight. The following morning the culture was diluted 20-fold with new TSB medium and incubated at 37 °C for a further 3 h to ensure that cells were in early exponential growth phase. These cultures were used for experiments by diluting to the desired concentration into donated human urine (at least 100-fold dilution with 0.2 μm filter-sterilized urine).

### Cell counts

The concentration of bacteria in the parent culture was counted using the Miles and Misra dilution and plating technique as described previously (Wand et al. 2015). This was done pre and post capture when evaluating capture efficiency of the anion-exchange beads, or only pre-capture when conducting titrations for DNA amplification. Several dilutions of the culture were prepared in Phosphate Buffered Saline (PBS) and, for every dilution, three 20 μL aliquots were dispensed onto duplicate TSA plates. Colonies were counted after an overnight incubation at 37 °C and these counts were used to infer the number of viable bacteria in the original culture, expressed in colony forming units (cfu).

### DNA extraction for control samples

DNA from an overnight culture of *Klebsiella pneumoniae* NCTC 13443 was extracted using the DNeasy Blood and Tissue Kit (Qiagen, UK) following the bacterial extraction protocol as described by the manufacturer. The eluted DNA was quantified on a Qubit® fluorometer using DNA assay reagents.

### Collection and characterization of urine samples

To evaluate our assay performance with urine samples of variable composition, we collected urine from different healthy volunteers. Samples were collected with ethical approval from the Southampton Research Biorespository Access Committee (Ref: 12/NW/0794). Approximately 50 mL of mid-stream urine was collected into a polypropylene tube and immediately filter sterilized using a Millex syringe filter (Millipore). The conductivity and pH of the urine samples were measured using a Horiba B-171 conductivity meter (Horiba, UK) and a pH indicator strip.

### Cell capture experiments

Bacteria were captured using 500 nm diameter magnetic beads coated with diethylaminoethyl (DEAE) (SiMAG-DEAE, 500 nm @ 7.5 × 10^11^ particles mL^−1^, Chemicell, USA) (Guo et al. 2009; Yang et al. 2011). The first step in the protocol was to establish the number of bacteria in the culture that adhered to the beads i.e. the efficiency of capture. A culture with an initial cell concentration of approximately 10^9^ cfu mL^−1^ was diluted 100 times with filtered urine to 10^7^ cfu mL^−1^. A 5 μl aliquot of DEAE bead stock suspension was added to the sample and mixed by gentle inversions until the solution appeared homogeneous. The samples were then incubated for 5 mins at room temperature without mixing to promote attachment of bacteria to the beads. The beads were removed from the solution by placing the tubes on a magnetic rack (Promega, UK). To estimate the capture efficiency, the concentration of bacteria in the initial culture (A) and the supernatant (B) (after removing magnetic beads) were compared. The per cent capture efficiency was calculated as 100-(B/A ×100).

### Sample preparation device

**Fig. 1:**
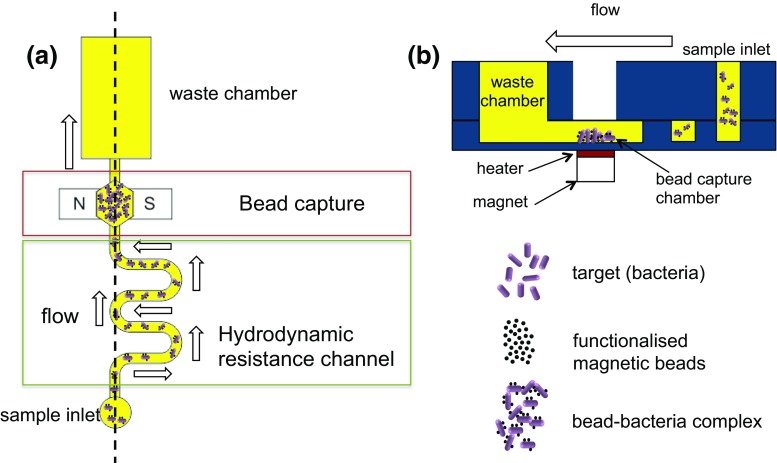
**a** Schematic representation of the sample preparation device showing a single channel. A long microfluidic serpentine channel controls the flow rate of a sample (bead-bacteria complexes in urine) pushed through the device with a pipette. This connects to a small volume bead capture chamber, placed above a pair of permanent magnets, and a 1 mL waste chamber (diagram not to scale). **b** Cross sectional view of the device along the *dotted line* shown in Fig. 1a. The chip is placed in a holder, which contains the magnets for bead-bacteria complex concentration and heater for thermal lysis of the bacteria

The operating principle of the bead capture device is shown in Fig. 1. The sample is the target organism (*Klebsiella pneumoniae*) coupled to magnetic nanoparticles (DEAE) and is pushed through the device using a pipette. The device consists of a long serpentine channel that provides a defined hydraulic resistance to control the flow rate of particles flowing through the magnetic bead capture chamber where the beads are captured. The urine sample minus the beads is collected in the waste chamber. A multichannel electronic pipette was used to deliver samples into eight parallel channels at a constant flow rate defined by the hydrodynamic resistance of the serpentine channel. For a given cross section, the flow rate of the sample across the magnets can be varied by changing the channel length. The capture chamber is located above a pair of bar-shaped permanent magnets separated by a distance of 1 mm (Fig. 1a), that are mounted in a base plate. The bead-bacteria complexes are captured in a chamber with a volume of approximately 6.5 μL that is closed with a thin layer of tape. After capture, the concentrated bead-bacteria complexes can either be removed from this chamber, or heated *in-situ* to lyse the bacteria and release the DNA using a resistive heater placed between the magnets and the device (Fig. 1b). The lysate containing DNA, lysed cells and magnetic beads in urine is retrieved for DNA amplification.

**Fig. 2:**
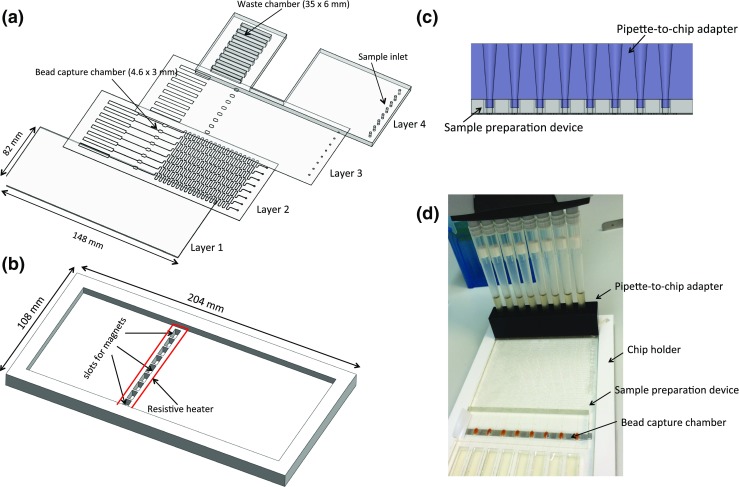
Diagram of **a** the sample preparation device assembly and **b** the holder. The chips is made of four PMMA layers machined using a CO_2_ laser. Layer 1 (thickness = 175 μm) forms the base of the device, layer 2 (175 μm PMMA + 2 × 50 μm of double sided tape on both sides) defines the channels (width ~140 μm) and bead capture chambers, layer 3 (175 μm) contains access holes for the bead capture chamber and seals the micro-channels. Layer 4 (5 mm) provides depth to the chip and holds the pipette-to-chip adapter. **b** the holder contains the permanent magnets placed in slots which are separated by a distance of 1 mm. *Red lines* in the figure show the position of the Kapton heater strip that is placed above the magnets. **c** Cross sectional diagram of the pipette-to-chip adapter that interfaces with an 8-channel electronic micropipette. The adapter is fabricated using a 3D printer from a rubber-like material and accommodates the conical pipette tips. **d** Photograph of the device showing the 8 channel pipette; bead-target complexes are immobilised in the chamber while supernatant is collected in the waste chamber

The microfluidic bead concentration device (8 channels) is mounted in a holder that holds the magnets and the resistive heater. The disposable microfluidic devices clips into the holder during operation. A schematic representation of the device assembly is shown in Fig. 2a. The device was fabricated from sheets of poly-(methyl methacrylate) (PMMA), which were cut using a CO_2_ laser (Epilog laser mini, USA). Layer 1 formed the base of the device; layer 2 contained sample inlets, microfluidic channels, capture chambers and the waste chambers; layer 3 sealed the channels while providing access to the capture chambers; and layer 4 provided depth to the chip. Layers 1, 2 and 3 were fabricated from thin sheets of PMMA (175 μm PMMA, Goodfellow, UK) while layer 4 was made from 5 mm thick PMMA (Techsoft, UK). First, the microfluidic channels (~140 μm width, 313 mm length), beads capture chambers (hexagon shape, 4.6 × 3 mm) and waste chambers (35 × 6 mm) were cut into 175 μm thick PMMA. This sheet was coated on both sides with double-sided tape (467 MP, 50 μm, 3 M) (layer 2). Layer 2 (148 × 82 mm) was then bonded to layer 1 (148 × 82 mm) and layer 3 (148 × 82 mm) to seal the microfluidic channels (Fig. 2a) whilst allowing access to the bead capture chamber. The PMMA stack (thickness = 625 μm) was bonded to a micromachined PMMA substrate (5 mm) with rectangular windows for access to the capture chambers.

The chip holder was fabricated from laser cut PMMA (Fig. 2b). The bottom layer (204 × 108 mm) of the holder contained 9 slots (8 × 4 mm) to hold the magnets (Neodymium, max operating temperature 100 °C, 48 M magnetisation strength, Supermagnet, Germany). A Kapton insulated flexible heater (1 × 10 cm, Omega, UK) was placed above the magnets to heat the chips. The heater was connected to a Proportional Integral-Derivative (PID) (EMKO, ESM-4420, Turkey) to maintain the chip temperature at 95 °C. The chip was fixed into the holder in such a way that each bead capture chamber was above a pair of magnets as shown in Fig. 1a. A pipette-to-chip adapter was made to house the conical shape pipette tips and fabricated using a 3D printer (Objet, Stratasys, USA). The adapter was made from a proprietary rubber-like material (FX9043) that provided a perfect fit for the pipette tips (Fig. 2c). It provided an easy, simple, and leak-free interface between the sample preparation chip and pipette. Prior to use, the capture chambers were closed using a clear, removable PCR film (Eppendorf, UK).

To operate the device, the chip was placed into the holder. A 1 mL urine sample (with beads) was pushed through the microfluidic channels at a constant flow rate using the 8-channel electronic pipette. The bead-bacteria complexes were captured in the chambers and the supernatant was collected in the waste chamber. The chip was then removed from the holder, the waste chambers emptied and the PCR film covering the capture chambers removed so that the beads (in 6.4 μL urine) could be recovered for further processing.

### Bacteria concentration on device and lysis off device

**Fig. 3:**
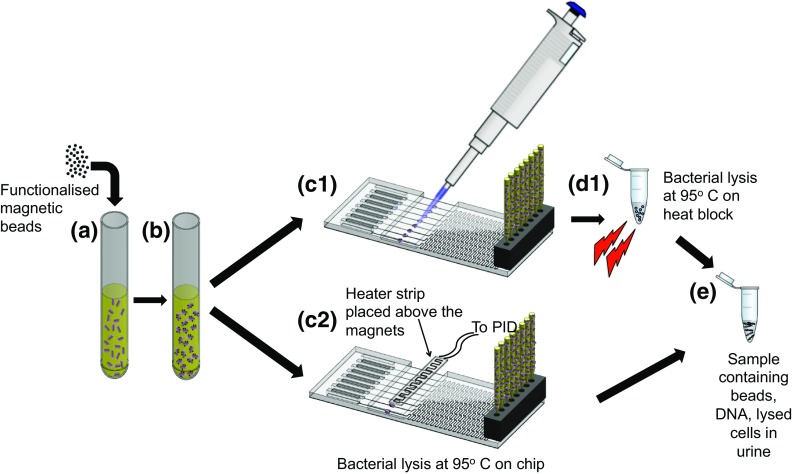
Schematic of the assay workflow: **a** DEAE functionalized magnetic beads are added to the urine. **b** The sample is incubated for 5 min at room temperature to promote attachment of the beads to the bacteria. **c** Sample is concentrated from 1 mL to 6.4 μL using the sample preparation device. (C1) The bacteria-bead complex can be aspirated from the bead capture chamber, transferred to a microcentrifuge tube and the collected sample is heated at 95 ° C on a heat block (D2). Alternatively, (C2) the sample is heated on the device using a resistive heater to a temperature of 95 °C to lyse the cells and release DNA. (E) Sample containing DNA, cell lysate, DEAE beads in urine is used for RPA reaction

A bacterial culture of *K. pneumoniae* with an initial concentration of approximately 10^8^ cfu mL^−1^ was diluted serially down to 10 cfu mL^−1^ with filter sterilized human urine. Each experiment consisted of triplicate dilution series and the experiment was repeated 3 times using urine from different volunteers. Figure 3 shows the workflow for the assay. Urine samples (1 mL) containing bacteria were spiked with 5 μl DEAE stock bead suspension and mixed by gentle inversions until the solution appeared homogeneous. The samples were then incubated for 5 mins at room temperature without mixing to promote attachment of bacteria to the beads. The sample was pushed through the device using the multichannel pipette at approximately 300 μL min^−1^ and the beads were concentrated into a volume of 6.4 μL. After removing the PCR film, the 6.4 μL sample was aspirated from the collection chambers and transferred to a clean 1.5 mL microcentrifuge tube. These tubes were placed immediately on a heat block and incubated at 95 °C for 5 mins to lyse the bacteria and release the DNA. The final crude lysate contained DNA, DEAE beads and lysed cells in the 6.4 μL of urine.

### Bacteria concentration and lysis on device

Cell lysis was also performed on device using the resistive film heater. The temperature in the chamber was measured using a K type thermocouple (RS components, UK) placed in one of the capture chambers. The heater was set to a target temperature of 95 °C. After heating the bead-bacteria complexes for 5 min, the chambers were allowed to cool (approximately 30 s) and the samples were recovered for DNA amplification by RPA.

### Real time recombinase polymerase amplification assay

Real time RPA was used to amplify the *bla*_CTX-M-15_ gene, using the TwistAmp® Exo kit. according to the manufacturer’s protocols. Lyophilised RPA proteins were reconstituted with a mix comprising rehydration solution, forward and reverse primers and sample. In each 50 μL reaction, the final concentrations of primers and FAM labelled probe were 0.48 μM and 0.12 μM, respectively. A 5 μl aliquot of the urine sample with the beads and lysed cells was added to this mix. Each RPA reaction mix was transferred to a well of a non-binding, black polystyrene 96-well plate (Corning, UK). The amplification reaction was initiated by adding magnesium acetate to a final concentration of 14 mM and mixing the reaction vigorously. The plate was transferred to a GloMax microplate reader (Promega, UK) set to 39 °C and the fluorescence measured at 1 min intervals for 40 min. The Time to Positivity (TTP) for each sample was measured from the time at which the fluorescence exceeds a threshold value equal to three times the standard deviation of the negative controls (urine with beads but no bacteria).

## Results and discussion

### Sample preparation device

Traditional bench-top protocols for DNA extraction from bacteria captured on magnetic beads require numerous pipetting steps and can introduce inaccuracies in the final sample volume due to various factors such as adhesion of sample to the microcentrifuge tubes and pipetting errors. Hence, the pre-concentration device was key to streamlining the sample preparation protocol, concentrating the pathogens into a defined sample volume that is ready for cell lysis and DNA amplification.

**Fig. 4:**
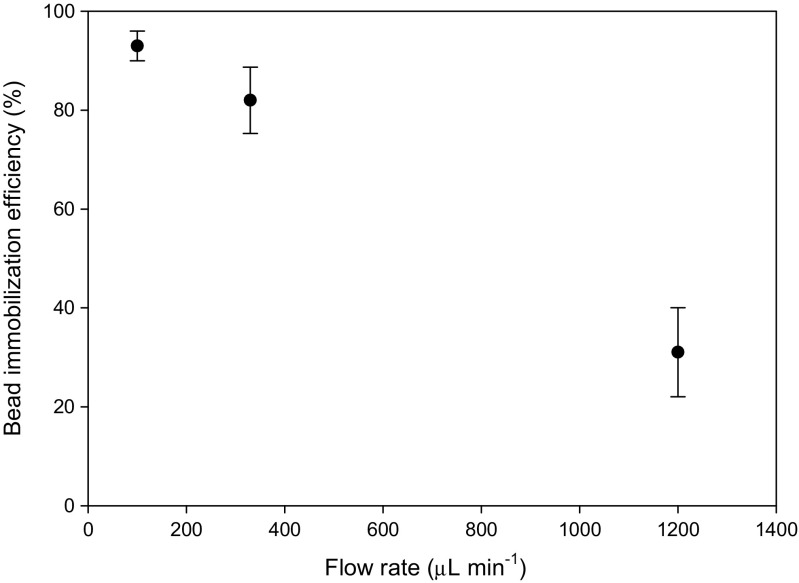
The percentage of beads captured in the chamber (bead immobilization efficiency) of the sample preparation device vs sample flow rate. Each data point is the average of immobilization efficiencies of the 8 channels on a single device (*n* = 1). *Error bars* represent S.D between the efficiency in the 8 channels. The flow rate was varied by changing the hydrodynamic resistance, hence the microfluidic channel dimension

The bead immobilization efficiency of the device was first measured as a function of flow rates. The electronic pipette provides a constant displacement so that the flow rate through the device depended on the hydrodynamic resistance of the channel. To determine the optimum flow rate, 5 μL of beads (7.5 × 10^11^ particles mL^−1^) were suspended in 1 mL of PBS and flowed through the device at different rates by varying the channel length to change the hydrodynamic resistance. Figure 4 shows the percentage of beads captured in the chamber for three different flow rates. The bead immobilization efficiency varied from 93 % at a flow rate of 100 μL min^−1^ (sample processing time = 10 min) to approximately 35 % for a flow rate of 1200 μL min^−1^ (sample processing time = < 1 min). A flow rate of approximately 300 μL min^−1^ was chosen as a compromise between a reasonable processing time and a high bead immobilization efficiency of 85 %. Using this flow rate, all 8 samples could be processed in parallel in approximately 3.5 min. The orientation of the magnets in the holder was also assessed. It was found that the best trapping efficiency was for N-S orientation, this dropped by 12 % for N-N (or S-S) orientation.

### Capture of bacteria on DEAE beads

**Fig. 5:**
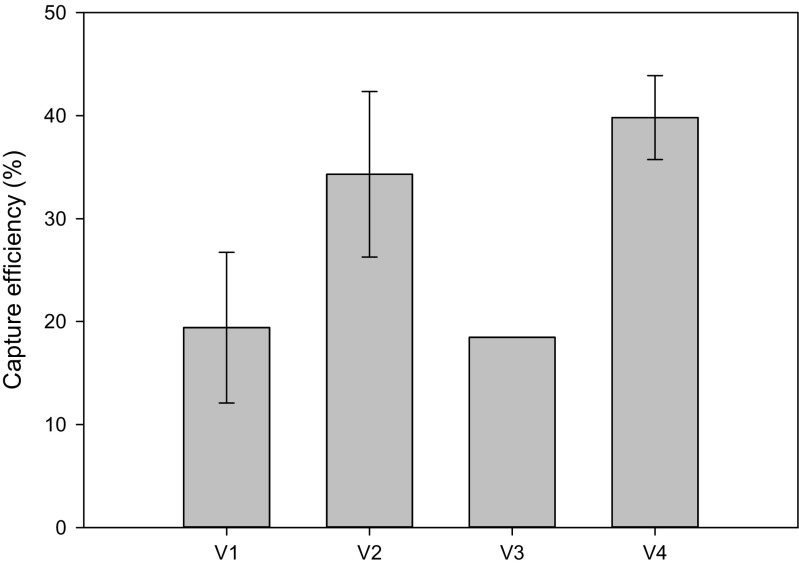
Average capture efficiency of *Klebsiella pneumoniae* NCTC 13443 (± standard deviation, *n* = 3) spiked into filtered urine from four healthy volunteers (V1-4), using anion - exchange DEAE magnetic beads. The *error bar* for V3 is too small to be depicted on the graph

The anion-exchange DEAE beads captured *Klebsiella pneumoniae* NCTC 13443 bacteria in human urine with a variable efficiency of between 18 and 39 % for an incubation time of 5 min, at a cell density of 10^7^ cfu mL^−1^ (Fig. 5). This density was used to mimic a typical heavily contaminated UTI sample. This is important to avoid the possibility of false negative tests failing to detect the presence of low numbers of ESBL gene-carrying bacteria, for example, in a mixed infection. Samples from four healthy volunteers showed significant variation in capture efficiency. Conductivity measurements were used to estimate the of salt concentration of the urine, and this ranged from 3.6 to 20 mS cm^−1^ with a mean of 12.5 mS cm^−1^ (Table S1) well within the normal range for human urine (Fazil Marickar 2010). The pH was also within the range that might be expected for healthy urine samples. There was no correlation between the conductivity and the efficiency of cell capture. The capture efficiency measured is in keeping with the range reported with other protocols in the literature, even when these are carried out in water or buffer solutions. For example, the capture efficiency of *E. coli* using DEAE magnetic beads was reported to be near 100 % (Yang et al. 2011), but the cells were suspended in H_2_O and incubated for 20 min. Other approaches using beads functionalized with antibodies (Zhu et al. 2011; Wen et al. 2013; Cho et al. 2014; Suh et al. 2014), aptamers (Suh et al. 2014) or vancomycin (Wang et al. 2014), generally require longer incubation times to achieve maximum capture efficiencies ranging from 2–98, 13 and 23 %, respectively. Additionally, these tests were only performed with bacteria suspended in either buffer or water. In contrast, our protocol is quick, does not require any bead conjugation and performs well with human urine.

### Amplification of the CTX-M gene in the presence of beads and urine

**Fig. 6:**
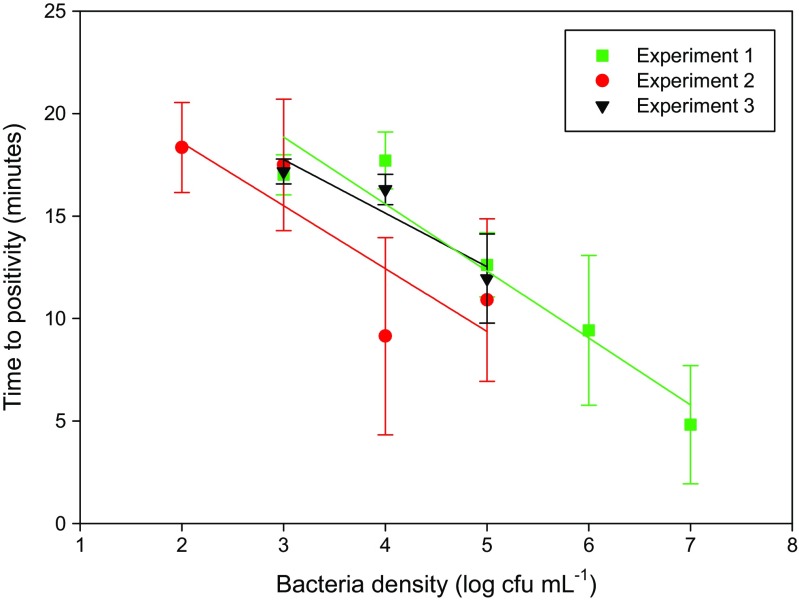
Plot of average time to positivity (TTP) of RPA reactions against the number of cells spiked into filtered human urine (*n* = 3). A linear regression is plotted for each experiment showing that the TTP is approximately proportional to the density of bacteria (Exp. 1 *R* ^2^ = 0.9218; Exp. 2 *R* ^2^ = 0.732; Exp. 3 *R* ^2^ = 0.8715). The limit of detection is at or below 1000 cells for Experiment 1 and 3, and 100 cells in Experiment 2

The RPA reaction performed well in the presence of DEAE beads and urine as evidenced by the steep rise in fluorescence during exponential amplification (Fig. 6; Figure S1) and a TTP that compared well with previous results for pure DNA (Kalsi et al. 2015). RPA has a high tolerance to impurities, meaning that nucleic acid isolation and purification is often not required, even for complex sample matrices such as human serum (Kersting et al. 2014a, b), goat pleural fluid (Liljander et al. 2015) and human urine (Krõlov et al. 2014). In this work we demonstrate amplification of bacterial DNA directly from heat lysed bacteria in human urine, as previously reported for *Chlamydia trachomatis* (Krolov et al. 2014). The RPA also works well in the presence of the magnetic nanoparticles, onto which the heat lysed bacterial cells, and possibly the released DNA, may remain attached. In conclusion, the RPA assay performs well using magnetic bead cell pre-concentration and unpurified DNA amplification from urine samples.

Samples of bacteria spiked into urine samples from three different volunteers (Table S1) showed a minimum reliable limit of detection (LoD) of 1000 cfu (total spiked in assay), with a TTP of approximately 17–18 mins (Fig. 6). This LoD is likely to be an underestimate as the actual number of cells captured by the beads is lower than initially spiked into the urine samples. As expected, the TTP decreases with increasing bacterial counts, indicating that the assay does not saturate at high cell numbers, within the ranges tested. A complicated urinary tract infection (UTI) is defined by a pathogen load of >10^5^ cfu mL^−1^ in women and 10^4^ cfu mL^−1^ in men, and for an uncomplicated UTI a bacterial count of 10^3^ cfu mL^−1^ is considered to be clinically relevant (Grabe et al. 2013). Our assay shows excellent performance in this range and, with a LoD of 1000 cfu from an initial 1 mL urine sample, is suitable for clinical diagnosis of antibiotic resistant pathogens in both complicated and uncomplicated UTIs.

Although the LoD of 1000 cfu in our assay is sufficient for a diagnostic test, it is higher than the usual detection limits for RPA of 10 gene copies using pure DNA (Piepenburg et al. 2006; Kalsi et al. 2015; Xia et al. 2015) or 10 bacterial pathogens lysed in urine, Krolov et al. (2014)). The higher LoD is partly due to a higher background signal when DEAE microparticles are present presumably as they scatter the light. Also, if the cell lysates are attached to the beads, the DNA might not be as easily accessible to the RPA primers as for a pure solution. Therefore, the assay is a compromise, sacrificing some sensitivity for simplicity and time, while maintaining a clinically relevant limit of detection.

**Fig. 7:**
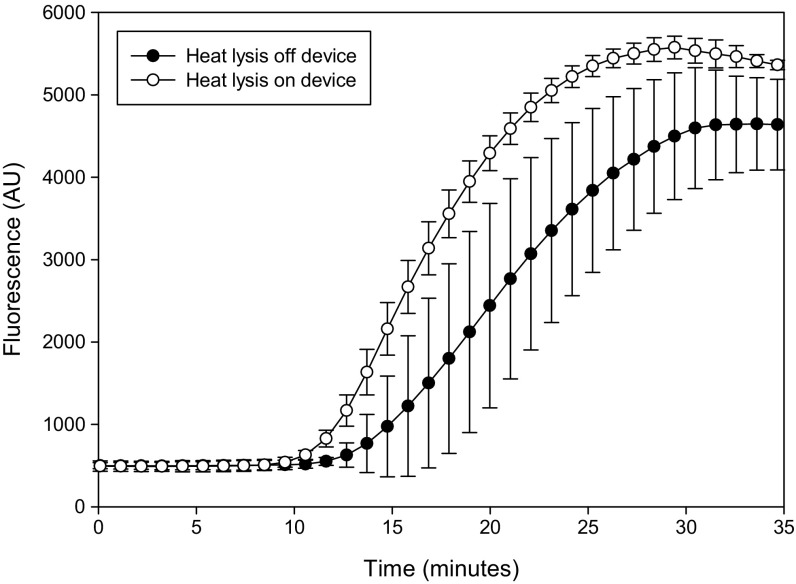
A comparison of average DNA amplification curves (± standard deviation, *n* = 3) for identical samples pre-concentrated on the sample preparation device and subjected to heat lysis either on the device or off the device in a microcentrifuge tube. The sample heated on the device shows faster amplification and a much higher reproducibility among replicates

**Fig. 8:**
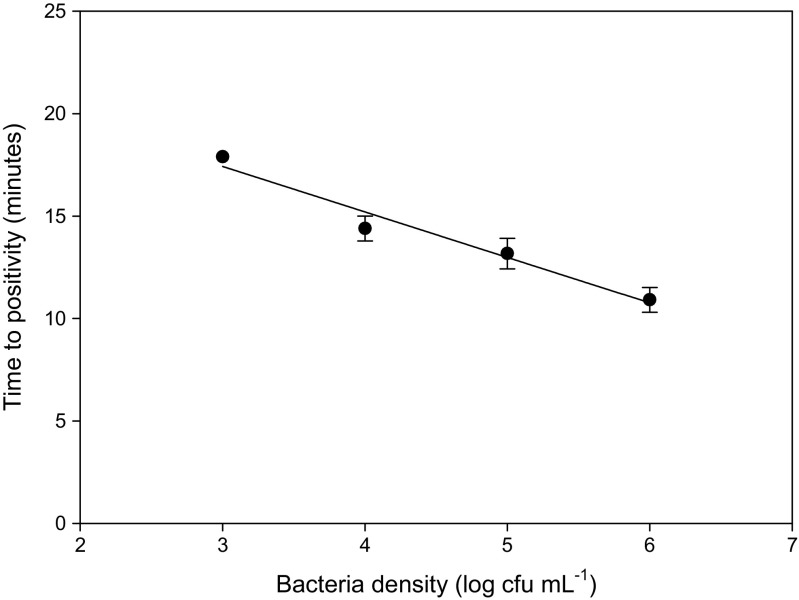
Data for an example end-to-end assay with heat lysis performed on the sample preparation device. Capture efficiency of bacteria, evaluated at 10^7^ cfu, was 26.6 %. The data is the average time to positivity (TTP) of reactions against the logarithm of the number of cells (*n* = 3). A linear regression shows that TTP is proportional to the density of bacteria (*R* ^2^ = 0.9636). The limit of detection is 1000 cells spiked into filtered human urine

Our simple single step sample preparation device, is able to reduce the sample volume from 1 mL to approximately 5 μL in a few minutes. The device also able to directly heat the sample to lyse cells. A comparison of RPA data for on-chip and off-chip heat lysis (Fig. 7) showed that there is a clear improvement in the TTP and a reduction in the S.D, probably due to reduction in losses from manual handling. The use of an integrated heater significantly improved the assay quality. *Klebsiella pneumoniae* spiked into urine at 10^7^ bacteria cfu showed a capture efficiency of 26.6 %, which is within the range obtained in the capture efficiency experiments shown in Fig. 5. A bacteria titration over a wide range of cell densities (10^3^-10^6^ cfu) was obtained on the device with integrated heater. This showed that the coefficient of variation (CV) of TTP in the RPA assay was lower than 5 % for all samples (Fig. 8), in contrast to the CV for samples heated off the device, where the CV ranged from 3.5 to 60 % in all previous experiments (mean = 22.5 %). This result confirmed the much higher reproducibility obtained when performing lysis on the device. It also confirmed the detection of ~266 captured cells (1000 spiked cells with capture efficiency of 26.6 %) with RPA in under 20 min. The final protocol takes approximately 45 min from sample to result, including 5 min incubation of the urine sample with beads, 3.5 min to concentrate the sample, 5 min for the heat lysis and 20 min for the RPA, with additional time (~12 min) for handling and RPA reagent preparation.

## Conclusions

We have demonstrated a simple device for capture and pre-concentration of bacteria spiked into human urine, followed by heat lysis for DNA amplification using RPA. The simple disposable device is easy to use and relies on the use of anion-exchange beads and DNA amplification from crude heat lysed cells, eliminating the need for centrifugation, bead functionalization and DNA purification. Sample handing and reproducibility is greatly improved by the use of on-chip heating. Cells can be pre-concentrated from 1 mL of urine within 3.5 min. Heat lysis of cells on the device followed by RPA delivers a simple integrated sample preparation system for processing a urine sample to amplification-ready DNA. We anticipate that the system and protocols developed could have widespread use in any application where bacteria need to be pre-concentrated from a liquid sample matrix using magnetic beads.

## Electronic supplementary material

ESM 1 (DOC 32 kb)
