# Supplementary material for: Simple and rapid sample preparation system for the molecular detection of antibiotic resistant pathogens in human urine
Source: Biomed Microdevices. 2016 Feb 4;18:18. doi: 10.1007/s10544-016-0031-9 (PMC4742488; doi:10.1007/s10544-016-0031-9)
Supplement: Supplementary file 1 — (DOC 32 kb) [file 10544_2016_31_MOESM1_ESM.doc]

**Supplementary information**

Table S1. Characteristics of filtered urine samples used in capture efficiency tests

| Experiment | Conductivity (mS/cm) | pH |
| --- | --- | --- |
| Capture efficiency tests (set A; Figure 3) | | |
| A1 | 9.5 | 7±0.5 |
| A2 | 11.1 | 7±0.5 |
| A3 | 20 | 7±0.5 |
| A4 | 3.6 | 7±0.5 |
| Initial RPA assays (set B; Figure 4) | | |
| B1 | 9 | 7±0.5 |
| B2 | 9 | 7±0.5 |
| B3 | 10.3 | 7±0.5 |
| Comparison of heating on or off device (C; Figure 5) | | |
| C1 | 15.2 | 7±0.5 |
| Final RPA assay after heating on device (D; Figure 6) | | |
| D1 | 3.6 | 7±0.5 |

Figure S1. A representative set of amplification curves from the end-to-end assay (Experiment 5; Table S1) showing the increase in fluorescence with time during the RPA reaction, for different cell concentrations. Initially, there is a lag phase where the fluorescence in the reaction is too low to be detected. Eventually, enough of the target DNA is amplified to produce a detectable fluorescence signal. The time where the fluorescence crosses the threshold value is the time to positivity. The threshold cannot be depicted as it is masked by the curves of the blank samples. In this experiment, the limit of detection is 1000 spiked cells.
